# Pulmonary Nocardiosis Diagnosed by Plasma Metagenomic Next‐Generation Sequencing in a Patient With Recurrent Febrile Neutropenia

**DOI:** 10.1155/crdi/2832247

**Published:** 2026-07-15

**Authors:** Maria Vega Brizneda, Jessica Lum, Hannah Wang, Zachary A. Yetmar

**Affiliations:** ^1^ Department of Infectious Disease, Cleveland Clinic, Cleveland, Ohio, USA, clevelandclinic.org; ^2^ Department of Pathology and Laboratory Medicine, Cleveland Clinic, Cleveland, Ohio, USA, clevelandclinic.org

**Keywords:** Karius, mNGS, neutropenia, *Nocardia farcinica*, nocardiosis

## Abstract

**Background:**

Nocardiosis disproportionately affects immunocompromised hosts. Early identification of *Nocardia* infections is critical as delays can lead to worse outcomes. Species such as *N. farcinica* are associated with increased risk of dissemination and resistance. Diagnosis of opportunistic infections in immunocompromised populations relies on culture, antigen, or serologic methods that often have limited sensitivity or specificity. Plasma microbial cell‐free DNA metagenomic next‐generation sequencing (mNGS) offers a noninvasive approach for early diagnosis of opportunistic infections.

**Case Presentation:**

We report a case of pulmonary nocardiosis in an 87‐year‐old man with myelodysplastic syndrome and prolonged neutropenia diagnosed by plasma mNGS. He had been hospitalized multiple times with recurrent febrile neutropenia and respiratory symptoms. Standard noninvasive microbiologic workup was unrevealing, but lower respiratory specimens could not be readily obtained. Due to elevated risk of complications from invasive testing, plasma mNGS was used as a complementary tool and identified *N. farcinica*. Anti‐*Nocardia* therapy was initiated, and his fevers resolved.

**Conclusion:**

mNGS is an emerging diagnostic tool that may identify *Nocardia* species from clinical specimens with a faster turnaround time than culture and enables rapid species identification. Although culture is still recommended for susceptibility testing, mNGS may expedite diagnosis in particular situations. This case supports the role of plasma mNGS as a complementary tool in the evaluation of febrile neutropenia and highlights its diagnostic potential.

## 1. Introduction

*Nocardia* is a genus of ubiquitous, partially acid‐fast bacteria that most commonly infect the respiratory system [1]. Nocardiosis disproportionately affects immunocompromised patients, such as those receiving immunosuppressive therapy or individuals with cancer [2]. These patients are often at risk for multiple infectious complications such as invasive fungal and viral infections and other opportunistic pathogens. These organisms present similarly but require different treatment, emphasizing the importance of establishing an accurate microbiologic diagnosis.

Diagnosis of opportunistic infections has traditionally relied on culture, antigen, or serologic methods, though these often have limited sensitivity or specificity, particularly in highly immunocompromised populations [3]. This can lead to delays in diagnosis, which have significant implications on outcomes. Molecular testing for pathogens, such as microbial cell‐free DNA metagenomic next‐generation sequencing (mNGS), may offer additional diagnostic value in these challenging cases [4–6]. We present a case of pulmonary nocardiosis diagnosed by plasma mNGS in a patient with myelodysplastic syndrome and prolonged neutropenia.

## 2. Case Presentation

An 87‐year‐old man presented to the emergency department with two days of worsening fatigue and one day of fever. He has a history of myelodysplastic syndrome, diagnosed approximately 5 years earlier after being found to have pancytopenia on routine laboratory evaluation. He was monitored with supportive care during this time, with a baseline absolute neutropenic count of about 0.5 × 10^9^/L. Due to a rising bone marrow blast count > 10%, chemotherapy was planned in the coming weeks, and he remained on prednisone 12.5 mg daily in the meantime. He resided in an urban setting and denied significant outdoor exposures or activities. He had a remote history of allergies to trimethoprim–sulfamethoxazole (TMP–SMX) and levofloxacin and was receiving acyclovir and cefdinir as antimicrobial prophylaxis. He was without significant respiratory or gastrointestinal symptoms. Chest X‐ray showed a mild left basilar infiltrate. Piperacillin–tazobactam 3.375 g IV every 6 h was started on admission with resolution of fevers. He was discharged after 5 days of antibiotic therapy.

He felt well until 3 days postdischarge when he developed recurrent fever, malaise, cough, and diarrhea. On readmission to this second hospitalization, he received piperacillin–tazobactam 3.375 g IV every 6 h. Chest X‐ray showed bibasilar opacities. Nasopharyngeal respiratory virus multiplex polymerase chain reaction (PCR) was negative. Stool multiplex PCR was positive for norovirus. He quickly defervesced and received 5 total days of piperacillin–tazobactam therapy before transitioning back to his usual prophylaxis with the addition of isavuconazole for mold prophylaxis and discharging from the hospital.

**FIGURE 1:**
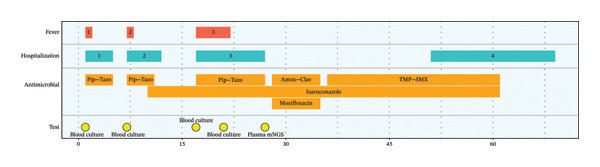
Timing of fever, hospitalization, antimicrobial therapy, and microbiologic testing. Abbreviations: Amox–Clav, amoxicillin–clavulanate; mNGS, metagenomic next‐generation sequencing; Pip–Tazo, piperacillin–tazobactam; TMP–SMX, trimethoprim–sulfamethoxazole.

The patient was hospitalized for a third time after presenting with fever, malaise, and cough 5 days after his last discharge. Chest X‐ray again showed bibasilar opacities, largely unchanged since his first hospitalization. Chest computed tomography showed bilateral lower lobe infiltrates with scattered nodules measuring up to 14 × 12 mm in the left upper lobe. Respiratory cultures were unable to be obtained as the patient was not producing sputum. Due to his recurring febrile neutropenia, noninvasive testing including blood cultures, nasopharyngeal multiplex PCR, urine *Histoplasma* antigen, plasma cytomegalovirus DNA, serum galactomannan, *Coxiella burnetii* antibodies, and serum *Cryptococcus* antigen were obtained and were all negative. He restarted piperacillin–tazobactam 3.375 g IV every 6 h and experienced fevers for several days after the date of admission. Due to his recurring febrile neutropenia and negative workup, plasma mNGS was sent during this hospitalization which utilized microbial cell‐free DNA isolation, extraction, and sequencing to compare results to a curated listed of > 1000 detectable human pathogens with high‐quality reference genomes (Karius Inc., Redwood City, California) [7, 8]. The patient was discharged while this test was pending, and it later resulted with *Nocardia farcinica* (123 molecules/100 nL). Due to his reported TMP–SMX allergy, he was initiated on amoxicillin–clavulanate and moxifloxacin based on prior literature [9]. Six days later, he underwent a TMP–SMX graded‐dose challenge without reaction, and his therapy was transitioned to TMP–SMX 10.7 mg/kg/day in two daily doses. Brain MRI did not show signs of central nervous system involvement, and he had no further fevers over the ensuing weeks. Approximately 1 month later, he was found to have progression of his myelodysplastic syndrome to acute myeloid leukemia. He was hospitalized and started decitabine, venetoclax, and cedazuridine therapy with poor tolerance. Soon after, he transitioned to hospice care and died without recurrent fevers or evidence of progressive infection (Figure 1).

## 3. Discussion

This case report highlights a patient who experienced recurrent febrile neutropenia due to initially unrecognized pulmonary nocardiosis, which was ultimately identified via plasma mNGS. The diagnosis of nocardiosis remains challenging due to the lack of standardized criteria, particularly from a pulmonary source [2]. Culture is the preferred diagnostic method; however, it has limitations such as the slow growing nature of the organism, which may require prolonged incubation (up to multiple weeks) and reduced sensitivity after previous exposure to antibiotics [1]. mNGS is an emerging diagnostic tool that uses sequencing of nucleic acid fragments and, in some cases, has shown advantages over conventional methods in the rapid detection of pathogens [6]. A targeted *Nocardia* PCR has been developed for respiratory samples. However, unlike pathogens such as *Aspergillus*, *Mucorales*, and other molds, *Nocardia* currently lacks a serum PCR. Plasma mNGS testing has been shown to identify *Nocardia* species from clinical specimens, leaving mNGS the only blood‐based diagnostic tool, though studies examining the clinical impact of mNGS testing for *Nocardia* specifically are lacking [8].

In immunocompromised hosts, early diagnosis is particularly valuable due to the increased risk for severe, disseminated, and rapidly progressive disease [10]. mNGS may provide a faster turnaround time than culture and enables rapid species identification, which is critical for guiding therapy due to the variable antimicrobial susceptibility profiles among *Nocardia* species [11]. Recent studies in febrile neutropenia have shown that plasma mNGS provides faster results than conventional testing and may improve diagnostic yield when results are interpreted by infectious diseases specialists [6]. A prospective, multicenter observational study evaluating the additive diagnostic value of mNGS in immunocompromised adults with pneumonia demonstrated that mNGS significantly increased the diagnostic yield, with one *Nocardia* case detected solely by mNGS and not by conventional testing [12]. In our patient, nocardiosis had not been identified through conventional methods, and mNGS enabled earlier diagnosis with the added benefit of avoiding an invasive procedure such as bronchoscopy. Given the potential for rapid clinical deterioration in disseminated or CNS nocardiosis, the ability to expedite diagnosis is a significant advantage. It is important to note that nocardiosis remains a rare diagnosis—in a review of 18,690 plasma mNGS reports, *Nocardia* species were identified in 69 (0.4%) cases [8]. Additionally, while not a usual *Nocardia* therapeutic, piperacillin–tazobactam does retain activity against some *Nocardia* isolates [13]. This may have accounted for temporary improvements in fevers and potentially impacted culture yield if invasive sampling was pursued.

The ability of mNGS to identify *Nocardia* to the species level, even for uncommon species such as *N*. *aobensis* and *N*. *otitidiscaviarum*, further illustrates its value, as species‐level identification has important prognostic implications [14, 15]. Certain *Nocardia* species, such as *N. farcinica*, are associated with higher rates of disseminated infection and other complications [16]. *Nocardia* also has species‐specific susceptibility profiles, and species identification can allow early tailoring of therapy [9, 17–19]. Nocardiosis is typically treated for prolonged periods, traditionally 6 months for pulmonary infection and ≥ 12 months for disseminated nocardiosis [2]. TMP–SMX, amikacin, imipenem, and linezolid are the antibiotics that *Nocardia* is most commonly susceptible to, though other agents such as amoxicillin–clavulanate, macrolides, fluoroquinolones, and tetracyclines can be therapeutic options [9, 17–19]. Molecular diagnostics do not provide formal antibiotic susceptibility data, and ideally *Nocardia* would still be isolated from culture for susceptibility testing due to the length of therapy and variability in pathogen‐specific susceptibility. In the present case, since no respiratory cultures for *Nocardia* were performed, therapy was selected empirically based on agents likely to be active against *N. farcinica*.

Despite its advantages, interpretation of mNGS results requires careful clinical correlation, as detection of *Nocardia* DNA does not always reflect infection. As with *Nocardia* PCR in respiratory samples, a positive result can reflect colonization rather than infection [20]. Therefore, expert review and integration of mNGS findings with clinical, radiologic, and laboratory data are imperative for diagnostic accuracy. However, this potential issue has not been well evaluated in nonrespiratory samples, such as plasma mNGS testing, and detection of *Nocardia* DNA from plasma may be more likely to reflect invasive disease.

## 4. Conclusion

mNGS is an emerging tool that may provide more rapid diagnosis of opportunistic infections such as *Nocardia* species, highlighting the potential advantages in cases with negative conventional results and/or high risks for invasive procedures. Additional research is needed to define populations benefiting from plasma mNGS testing; thus far this method may complement but is unlikely to replace conventional microbiology diagnostics. Infectious disease expertise is essential in the effective use and interpretation of this novel diagnostic tool.

## Author Contributions

Maria Vega Brizneda and Zachary A. Yetmar: writing–original draft, writing–review and editing, and conceptualization. Jessica Lum and Hannah Wang: writing–review and editing and conceptualization.

## Funding

No funding was utilized in the preparation of this manuscript.

## Ethics Statement

Institutional review board approval was not required for this case report in accordance with institutional policies.

No written consent has been obtained as there are no patient identifiable data in this report. All information has been sufficiently deidentified.

## Conflicts of Interest

The authors declare no conflicts of interest.

## Supporting Information

Additional supporting information can be found online in the Supporting Information section.

## Supporting information


**Supporting Information** *Nocardia* mNGS Case_CARE Checklist.

## Data Availability

Data supporting this work are available from the corresponding author upon reasonable request.
