# Supplementary material for: Pulmonary Nocardiosis Diagnosed by Plasma Metagenomic Next‐Generation Sequencing in a Patient With Recurrent Febrile Neutropenia
Source: Case Rep Infect Dis. 2026 Jul 15;2026:2832247. doi: 10.1155/crdi/2832247 (PMC13373437; doi:10.1155/crdi/2832247)
Supplement: Supplementary file 1 — Supporting Information Nocardia mNGS Case_CARE Checklist. [file CRDI-2026-2832247-s001.pdf]

CARE
case report guidelines

CARE Checklist of information to include when writing a case report

(cc) BY-NC-ND

Topic	Item	Checklist item description	Reported on Line
**Title**	1	The diagnosis or intervention of primary focus followed by the words "case report"	1
**Key Words**	2	2 to 5 key words that identify diagnoses or interventions in this case report, including "case report"	10
**Abstract (no references)**	3a	Introduction: What is unique about this case and what does it add to the scientific literature?	19-20
	3b	Main symptoms and/or important clinical findings	22-25
	3c	The main diagnoses, therapeutic interventions, and outcomes	25-27
	3d	Conclusion—What is the main "take-away" lesson(s) from this case?	28-32
**Introduction**	4	One or two paragraphs summarizing why this case is unique (**may include references**)	34-46
**Patient Information**	5a	De-identified patient specific information.	48-55
	5b	Primary concerns and symptoms of the patient.	60-61
	5c	Medical, family, and psycho-social history including relevant genetic information	49-51
	5d	Relevant past interventions with outcomes	58-66
**Clinical Findings**	6	Describe significant physical examination (PE) and important clinical findings.	57-58
**Timeline**	7	Historical and current information from this episode of care organized as a timeline	Figure 1
**Diagnostic Assessment**	8a	Diagnostic testing (such as PE, laboratory testing, imaging, surveys).	70-73
	8b	Diagnostic challenges (such as access to testing, financial, or cultural)	69-70
	8c	Diagnosis (including other diagnoses considered)	78-79
	8d	Prognosis (such as staging in oncology) where applicable	85-87
**Therapeutic Intervention**	9a	Types of therapeutic intervention (such as pharmacologic, surgical, preventive, self-care)	80-83
	9b	Administration of therapeutic intervention (such as dosage, strength, duration)	80-83
	9c	Changes in therapeutic intervention (with rationale)	80-83
**Follow-up and Outcomes**	10a	Clinician and patient-assessed outcomes (if available)	83-88
	10b	Important follow-up diagnostic and other test results	83-84
	10c	Intervention adherence and tolerability (How was this assessed?)	80-84
	10d	Adverse and unanticipated events	84-88
**Discussion**	11a	A scientific discussion of the strengths AND limitations associated with this case report	90-139
	11b	Discussion of the relevant medical literature **with references**.	90-139
	11c	The scientific rationale for any conclusions (including assessment of possible causes)	141-146
	11d	The primary "take-away" lessons of this case report (without references) in a one paragraph conclusion	141-146
**Patient Perspective**	12	The patient should share their perspective in one to two paragraphs on the treatment(s) they received	NA
**Informed Consent**	13	Did the patient give informed consent? Please provide if requested	Yes ☐ No ☒
